# Embankment deformation characteristics analysis of an expressway widening project near a pond

**DOI:** 10.1038/s41598-022-24592-w

**Published:** 2023-01-13

**Authors:** Chunwei Wu, Dufeng Zhang, Han Xia, Junhui Luo, Haifeng Huang, Da Qin

**Affiliations:** 1 Guangxi Beitou Transportation Maintenance Technology Group Co., LTD., Nanning, 530201 China; 2 Guangxi Road Construction Engineering Group Co., LTD., Nanning, 530010 China; 3 Jiangsu Key Laboratory of Urban Underground Engineering & Environmental Safety, School of Transportation, Southeast University, Nanjing, 211189 China; 4 Guangxi Communications Design Group Co., LTD., Nanning, 530029 China

**Keywords:** Civil engineering, Environmental impact

## Abstract

New and old subgrade stability is a crucial problem for widening projects in soft soil foundations because uncoordinated deformations can cause serious engineering accidents, even disasters. In order to ensure the stability of a widening expressway project near a pond in the Qinbei section, a series of on-site investigations, lab tests, and numerical analyses was performed. The settlement and displacement of on-site monitoring were carried out during the construction period to verify the analyzed results. It was found that the uneven settlement caused heavy settlement and displacement of the embankment; thus, net cracks and longitudinal cracks occurred in the expressway. The new embankment was also affected by the weak foundation; therefore, the foundation should be reinforced before the construction of the widening part. Considering the economy and effectiveness of the widening project, the replacement method was recommended for the weak foundation reinforcement based on the numerical analysis and on-site motoring results. Water pumping had a significant effect on the settlement of the embankment. Water pumping caused seepage in the foundation and increased the effective stress of the soil, making the foundation more consolidated. The pond slope should be reinforced before the construction of the embankment because it was not confined by the surrounding soil. Therefore, the slope soil could not provide sufficient passive soil pressure and easily slide.

## Introduction

Traffic growth is strongly correlated to the economy^1^. The development of the traffic system can stimulate goods exchange and generate more job opportunities. In addition, economic growth can spur investments in traffic construction. The Lanhai Expressway (G75) was operated in 2010 to create strong communications between the northern and southern regions of China. With the development of the Guangxi Beibu Gulf Economic Zone^2,3^ and the increase of communications with other countries, traffic jams and road problems started to occur frequently on the Lanhai expressway^4,5^. The construction of a new expressway is a time-consuming project and requires huge money; hence, expressway widening can be a more feasible approach^6–9^. The expressway widening approach can make good use of land sources and also save money and time. For example, the Guangfo expressway was constructed in 1997 and finished in 1999, and this project required nearly one year less as compared to its original construction.

Generally, expressway widening is executed in three methods—integrated widening, separated widening, and hybrid widening^10^. Integrated widening can be divided into unilateral, bilateral, and internal widening (Table 1). In the internal widening method, the median strip is transferred to a vehicle lane. Moreover, the internal widening method has low disturbance and can be easily constructed; however, in this case, widening areas are small. Separated widening can be divided into unilateral and bilateral widening. The hybrid widening method combines the characteristics of integrated and separated widening.

**Table 1:** Different types of expressway widening^10^.

Category		Widening form
Integrated widening	Unilateral widening	
	Bilateral widening	
	Internal widening	
Separated widening	Unilateral widening	
	Bilateral widening	
Hybrid widening	–	

Although the expressway widening approach is useful and economic, it faces some critical issues during design, construction, and operation periods. During expressway widening, long cracks, net cracks, and uneven settlement generally occur in original expressways due to weak pavements or foundations^11–13^. Furthermore, the uncoordinated deformations of new and old subgrades greatly affect widened parts of expressways^14–18^. As old subgrades are generally operated for many years and foundations become consolidated and deformed under their self-weight as well as under vehicle and vibration loads, foundations and old subgrades become stable. However, during widening projects, new embankment constructions can bear additional loads and gradually settle. However, new subgrades cannot settle under vehicle and vibration loads; hence, cracks appear at the boundary of new and old subgrades.

In order to solve this problem of inconsistent settlement, model tests^19,20^ and numerical analyses^21–24^ have been carried out to evaluate the deformation of widened expressways. Generally, inconsistent settlements are induced by soils^25–30^ and water^31^. The reduction of the underground water level decreases the pore pressure in soils and, consequently, increases the effective stress of soils^32–37^, leading to soil consolidation and settlement; thus, foundations significantly deform. Therefore, foundation reinforcement is necessary to avoid this problem. Foundation reinforcement is carried out through the following methods: (a) physical and chemical routes are used to consolidate new foundations directly, (b) composite foundations or piles are used to increase the rigidity of new foundations, and (c) new materials are used to fill embankments^38–41^.

The Qinbei expressway is a section of the Lanhai expressway, and it is one of the main channels to connect the southern cities of Guangxi in Qinzhou to Beihai. However, in recent years, the booming traffic has created heavy pressure on the Qinbei expressway. In order to decrease the expressway widening project cost, the unilateral widening method of the integrated widening approach has been adopted. However, the Qinbei section is located in the soft soil area of southern Guangxi, and some widening parts are crossing large soft soil layers. Especially in the K2119 + 550 − K2119 + 700 section, a pond near the embankment supplies sufficient water to the pores of the weak foundation. The present work focused on the soft soil foundation in the southern Guangxi province. Considering the influences of underground water, the effects of the replacement method on the stability of the widened embankment were explored. Finally, the deformation characteristics of the new and old embankments were discussed.

## Geology engineering conditions and project description

### Engineering geology

The expressway widening project is carried out in the Qingzhou and Beihai cities of southern Guangxi with three deposits: Alluvial-pluvial deposit, Colluvial deposit, and Residual deposit. The soil layers are as follows: The alluvial-pluvial deposit layer was composed of clay, silt, fine sand, medium sand, and coarse sand. The alluvial-pluvial deposit layer depth was about 1–3 m and contained organic matter. Next, the residual deposit layer comprised clay, sand, and gravel. The depth of the residual deposit layer was about 0–3 m, and it was in a hard plastic state. Finally, the colluvial deposit layer comprised clay, silt, fine sand, medium sand, and coarse sand. The depth of the colluvial deposit layer was about 3 m, and it was in a plastic state.

**Figure 1:**
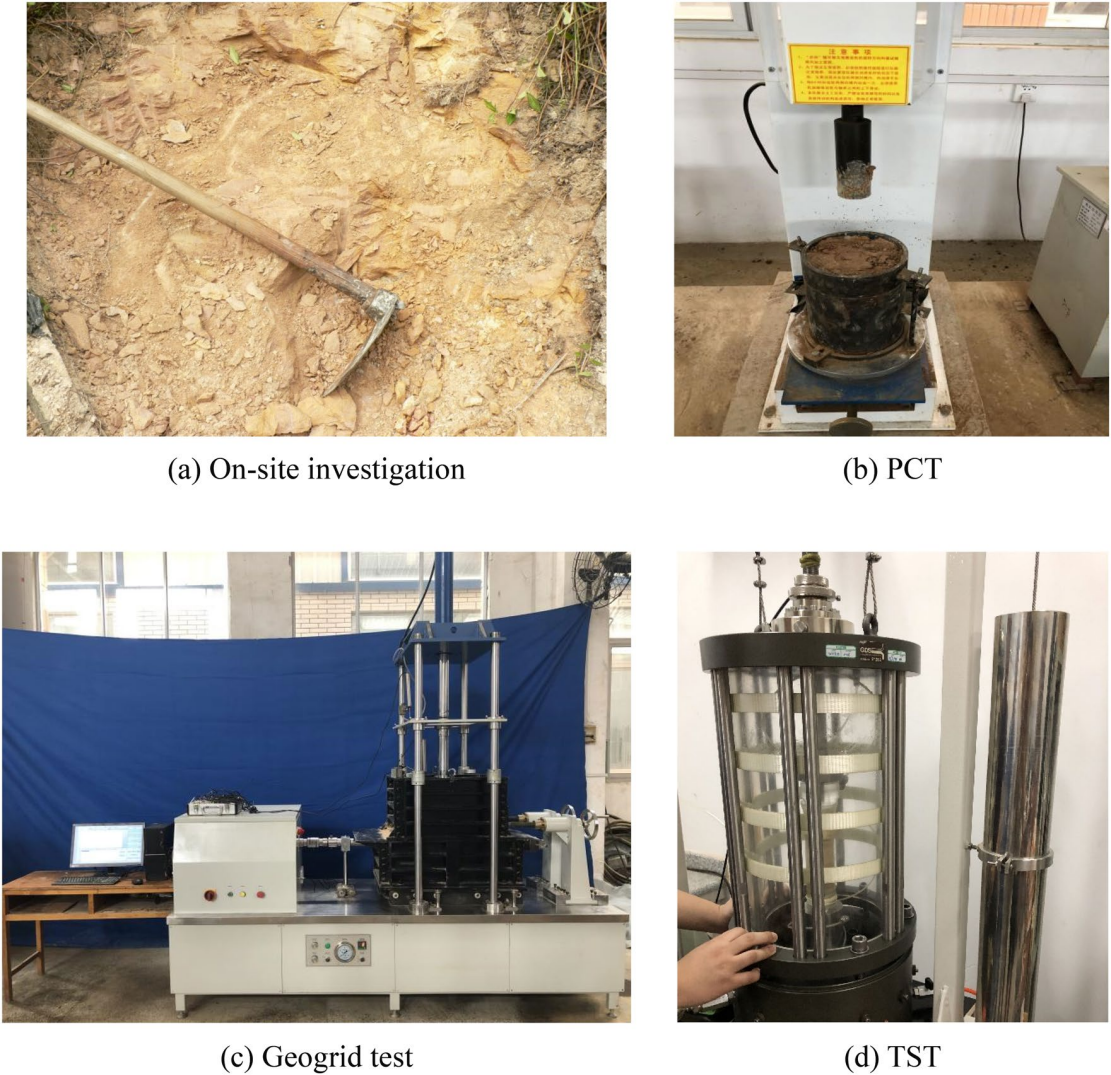
Different test processes.

In order to obtain the mechanical parameters of rocks and soils, a series of tests, such as direct shear test (DST), triaxial compression test (TST), standard penetration test (SPT), dynamic penetration test (DPT), California bearing ratio test (CBT), and proctor compaction test (PCT), was executed according to the Chinese GB 50021–2009 standard^42^. The sample quantities of the investigation are illustrated in Table 2, and the different test processes process is displayed in Fig. 1. The soft soil parameters are listed in Table 3.

**Table 2:** Sample quantities of the investigation.

Method	Sample	Unit	Quantity
Drill hole test	Bridge	m	6514
	Road	m	260
In-situ test	SPT	–	91
	DPT	m	29.6
Lab Test	Soil	–	34
	Rock	–	188
	Water	–	30

**Table 3:** Parameters of the weak foundation along the widened expressway.

Soil	*w* (%)	*ρ* (g/cm^3^)	*G*_s_	*e*	*w*_L_ (%)	*I*_P_	*a*_1-2_ (MPa^−1^)	*φ* (^°^)	*c* (kPa)	*f*_k_ (kPa)
Muck	61.0–87.5	1.50–1.64	2.68–2.70	17.1–2.23	51.5–59.3	–	–	–	–	–
Mucky clay	38.4–33.0	1.72–1.86	2.68–2.72	1.23–1.53	35.0–41.5	14.2–17.8	0.15–0.23	5.0–8.5	9.0–15.0	40–50
Silty clay	28.5–33.0	1.87–1.95	2.71–2.72	0.85–0.95	30.6–34.0	12.2–14.3	0.10–0.15	16–20	15.0–25.0	100–150

### Project description

The Qinbei expressway is an essential part of the Lanhai Expressway (G75), and it connects the southern area of Guangxi to Beihai. The Qinbei expressway starts from Gaoqiao county and ends in the Maowei sea, and its total length is 51.96 km. The original expressway was built in 2010, and it is a two-way four-lane expressway with an embankment width of 28.0 m. The embankment has a median width (central anti-collision wall) of 3.2 m, a carriageway of 4 m × 3.75 m, a hard shoulder of 2 m × 4.0 m, and a soil shoulder of 2 m × 0.9 m. The unilateral widening method of the integrated widening approach was adopted for this planned project, and the designed speed was 120 km/h. After the completion of the widening project, the K2119 + 550–K2119 + 700 section will become a two-way eight-lane expressway and significantly increase the transportation volume.

**Figure 2:**
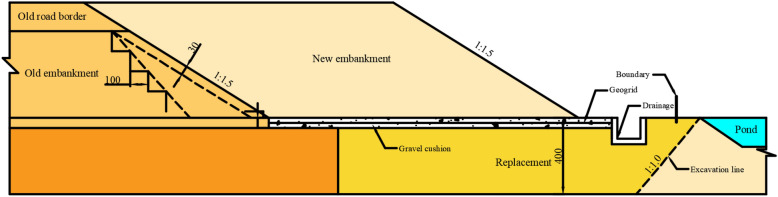
Layout of the widening project.

The K2119 + 550–K2119 + 700 section has a weak foundation area. The surface width of the new embankment is 33.0 m, the embankment slope ratio is 1:1.5, and the filling height is 10.3 m. The soft foundation has a depth of about 4.0 m and is covered with a silty clay soil layer. A pond is located near the expressway, and it is about 35.0 m wide, 42.0 m long, and 4.5 m deep. The new embankment is schematically illustrated in Fig. 2. The DPT results revealed that the soft soil had 13 hits, 17 hits, 17 hits, 17 hits, 20 hits, 25 hits, 32 hits, 36 hits, and 50 hits in the widened part near the pond along the depth. According to the recommended calculation method by the Guangdong Architectural Design & Research Institute^43^, the bearing capacities of the soft soil were determined as 82 kPa, 100 kPa, 100 kPa, 100 kPa, and 114 kPa along the depth; thus, it could not meet the designed bearing capacity. Therefore, the soft soil was treated before the construction of the foundation.

## Distress formation investigation and analysis

### Distress formation investigation

#### Original embankment

**Figure 3:**
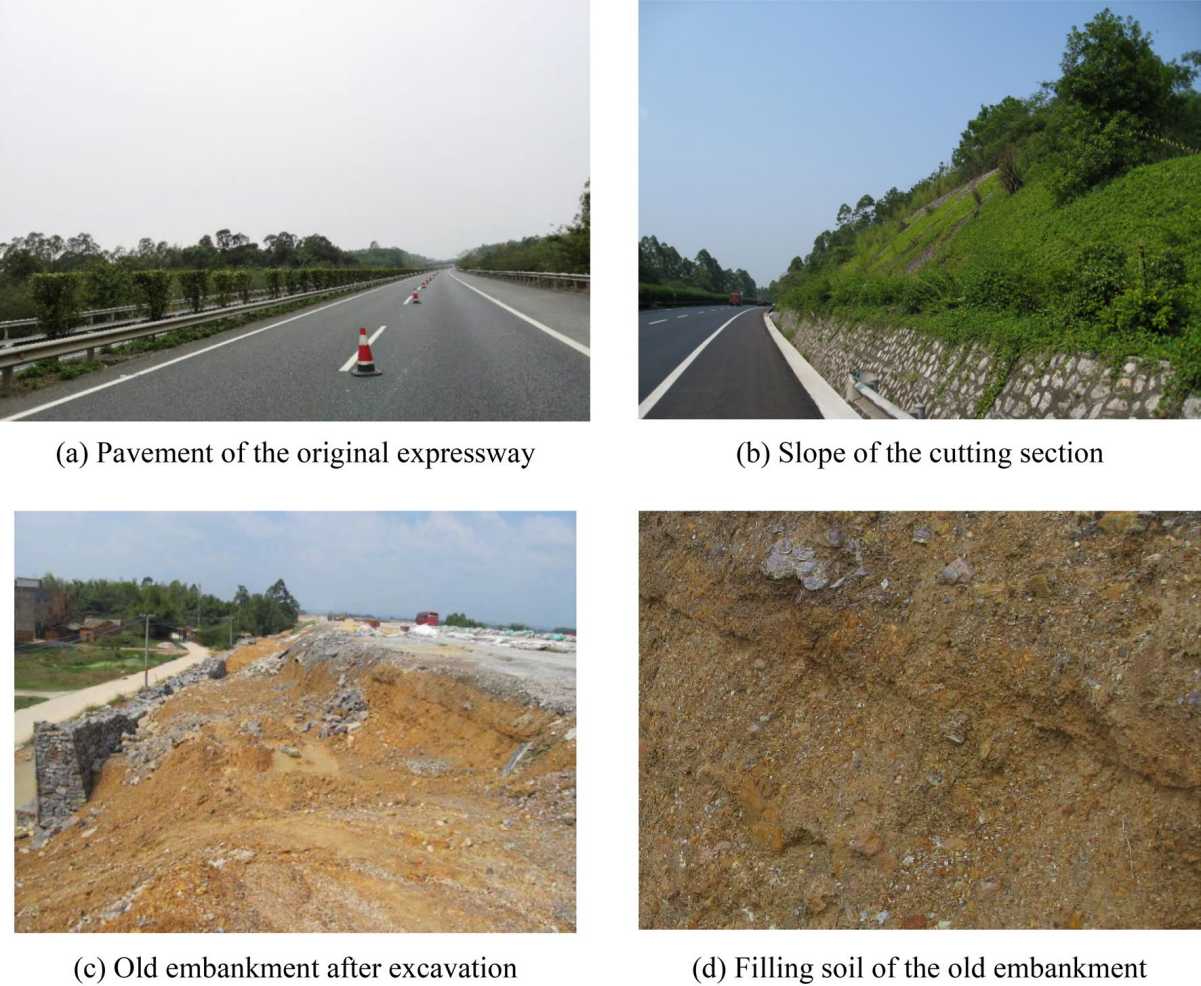
Original expressway.

The original embankment consists of an artificial filling layer and an underlying quaternary stratum (Fig. 3). The filling height ranges between 0 and 20 m. The filling slope has three steps, and each step has a height of 8 m and a width of 1.5 m. The slope rates for 1–3 step are 1:1.5, 1:1.75, and 1:2 from top to bottom. The embankment slope of the original expressway is low and gentle, and the slope height is mostly below 20 m. Vehicle jumping, pavement cracking, and abutment staggering occur in the old expressway because of the settlement of the embankment bridgehead. The compactness of the filling behind the abutment is low; therefore, the deformation and damage of the pavement occur in the transition section.

#### Pavement damage

**Figure 4:**
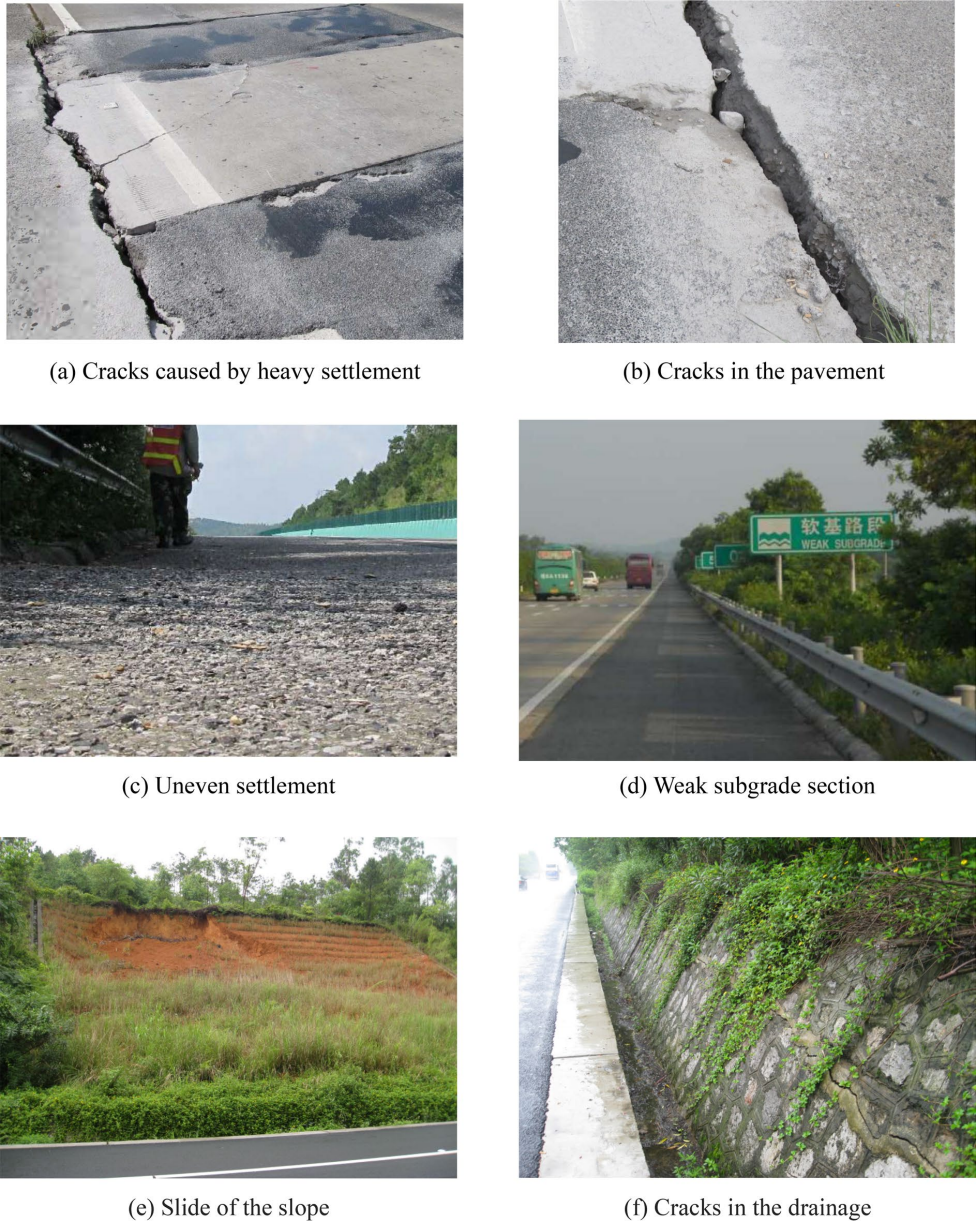
Distress formation of the old expressway.

After more than 10 years of operation, numerous damages have occurred in some sections of the pavement (Fig. 4). Although the expressway has been repaired every year, pavement damages, such as partial drop-offs, net cracks, and longitudinal cracks, frequently occur due to the increasing traffic volume. These damages have mainly occurred in the filling and excavation junction section or the high-filling part of the embankment section due to the uneven settlement of the embankment or the insufficient strength of the pavement base.

### Distress formation analysis

The pavement base is composed of lime fly ash-stabilized macadam. However, it is reported that lime fly ash-stabilized macadam has some disadvantages as compared to cement-stabilized macadam. For example, the surface of lime fly ash-stabilized macadam bases becomes soft due to water intrusion more quickly than that of cement-stabilized pavements. Cracks appear in cement-stabilized pavements due to the lack of a complete internal drainage system. The fine particle content in the lime fly ash gravel layer of the old pavement is high, the water scouring resistance is low, and water stability is poor.

The old pavement has been operated for a long time, and its strength is insufficient; thus, damages easily occur in the excavation junction section and the high-filling area of the embankment section. The widening project has adopted the unilateral widening method of the integrated widening approach to reduce the uncoordinated deformations of the old and new embankments. Therefore, this project should make a proper embankment splicing design.

## Numerical analysis

### Model description

**Figure 5:**
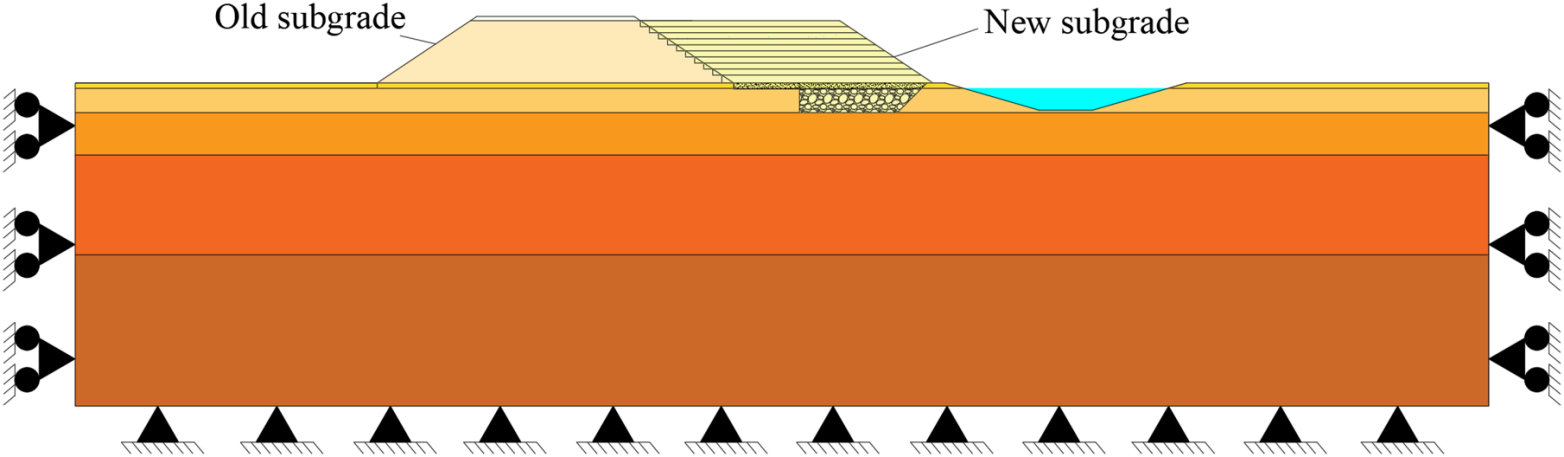
Layout of the numerical model.

A two-dimension numerical model of the proposed embankment was developed in ABAQUS software, and the model size and dimensions were consistent with those of the actual widening project (Fig. 5). The stratum model had a length of 234 m and a width of 53.4 m. The widths of the top and bottom of the embankment were 61.1 m and 92.0 m, respectively, and the width of the newly filled embankment was 33.0 m. The filling height was 10.3 m, the slope ratio was 1:1.5, and the replacement depth of the soft foundation was 4.9 m with a sand cushion of 1.0 m. For the convenience of calculations, the length and width of the pond were rounded. The pond was 2.0 m away from the new embankment and had a width of 40.0 m and a depth of 4.5 m. The modified Cam-Clay model was used to simulate the mucky layer, and the Mohr–Coulomb model was adopted to simulate the other layers. The soil element type was CPE4R. Thirteen analysis steps with a mesh of 26,199 elements were used to evaluate the stability of embankment slopes.

The following assumptions were made during the construction of the numerical model: (i) The old embankment and all soil layers were homogeneous and isotropic and (ii) the soft foundation replaced with gravel and the sand cushion were also homogeneous and isotropic. Moreover, the following boundary conditions were applied: (i) the bottom of the model was fixed, (ii) the left and right boundaries were horizontal and fixed, and (iii) the upper surface was free.

### Model parameters and analysis conditions

The physical and mechanical parameters of each soil layer of the numerical model are presented in Table 4. In order to analyze the effects of underground water, filling materials, and the replacement method on the stability of the embankment, two different cases were considered for numerical modeling: Case A: Step 1: The soft foundation was replaced with gravel. Step 2: The sand cushion was laid, and the embankment was filled. Step 3: Water was pumped from the pond. Case B: Step 1: The weak foundation was replaced with gravel. Step 2: The sand cushion was laid, and the embankment was filled.

**Table 4:** Parameters of the numerical model.

Layer	Thickness (m)	*c* (kPa)	*φ* (^°^)	*E*_s_ (MPa)	*µ*	*γ* (kN/m^3^)
Filled plain	0.8	12.0	8.0	5.0	0.4	17.7
Muck	3.0	4.6	6.0	3.76	0.47	17.8
Completely weathered sandstones	3.0–5.0	27.0	20.3	500.0	0.38	18.3
Strongly weathered sandstones	3.0–5.0	35.0	30.0	12.5 × 10^3^	0.3	19.9
Moderately weathered sandstones	25.0	40.0	35.0	25.5 × 10^3^	0.25	27.0
New embankment	–	32.0	22.0	25	0.3	19.0
Sand cushion	0.5	1	35.0	20.0	0.3	21.0
Old embankment	–	35.0	25.0	30.0	0.3	20.0
Old pavement base	–	–	–	1200.0	0.2	23.5
Crushed stones	3.3 m	–	–	300.0	0.19	27.5

### Numerical analysis results

#### Settlement of the embankment

**Figure 6:**
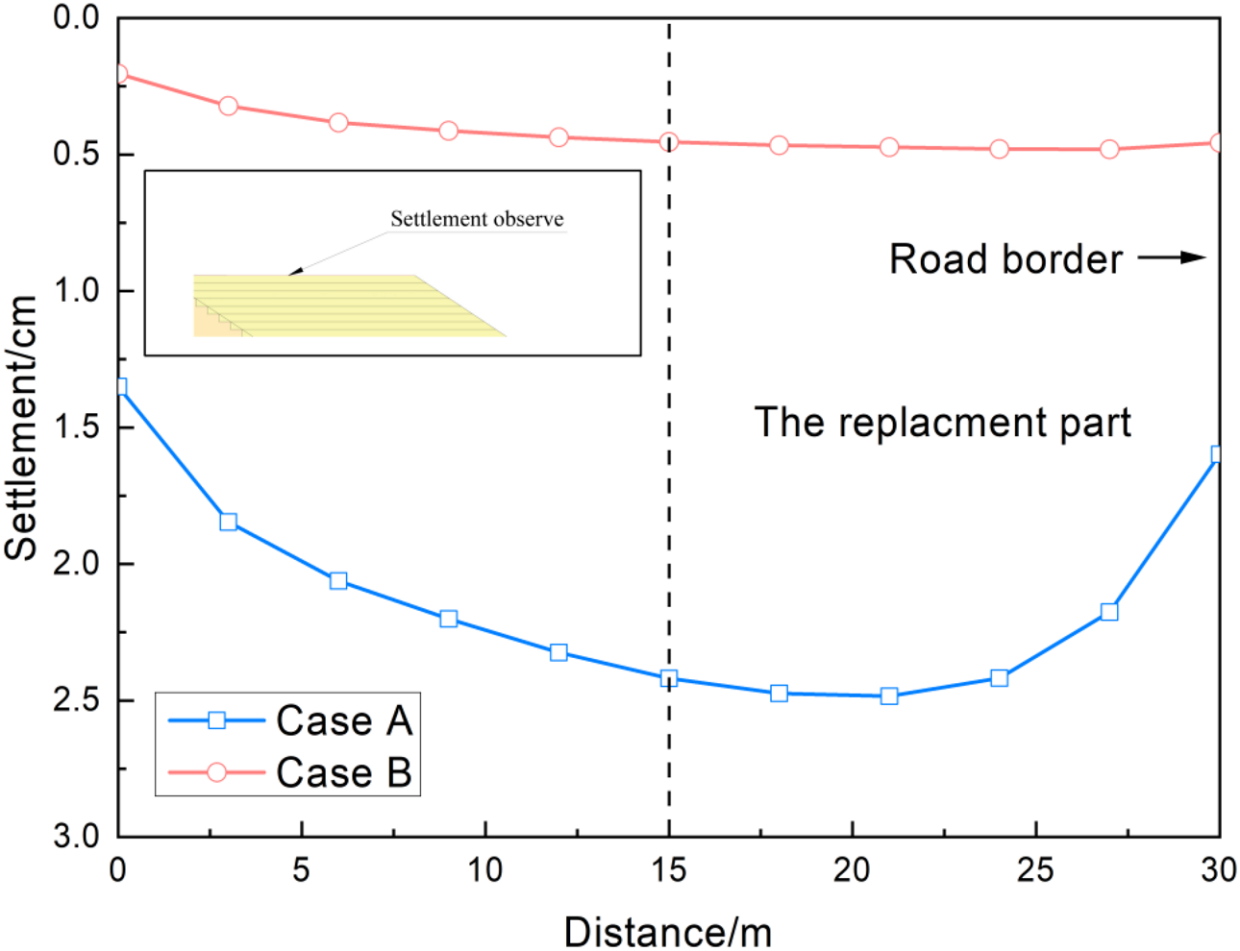
Final settlement of the embankment surface.

The final settlement along the new embankment at the depth of 1 m is displayed in Fig. 6. It is noticeable that after the widening of the embankment, the new embankment settled gradually. In comparison to the other parts of the embankment, the settlement of the replaced part was more prominent. At the same level of embankment settlement, the settlement increased with the distance closer to the replaced part. The maximum settlement in Case B was 0.46 cm, whereas the value in Case A was 2.48 cm. Although low-compressible materials were used to replace crushed stones, the consolidation of the foundation under the new permanent load continued; hence, the foundation gradually became consolidated and stable.

It is evident from the embankment settlement curves under the pumped and non-pumped conditions that the pumping method had a significant effect on the settlement of the embankment. The maximum and minimum differences were 4.01 cm and 1.14 cm, respectively, indicating that groundwater also significantly affected the stability of the embankment. In addition, the settlement difference between the replaced part and other parts of the embankment was profound, implying that water pumping greatly influenced the settlement of the replaced area. After water pumping, the new embankment provided a more permanent load to the foundation and the underground water level decreased due to seepage. According to the effective stress principle, the excess pore pressure dissipated and the effective stress increased; thus, the soil structure got compressed, leading to embankment settlement.

#### Displacement of the embankment slope

**Figure 7:**
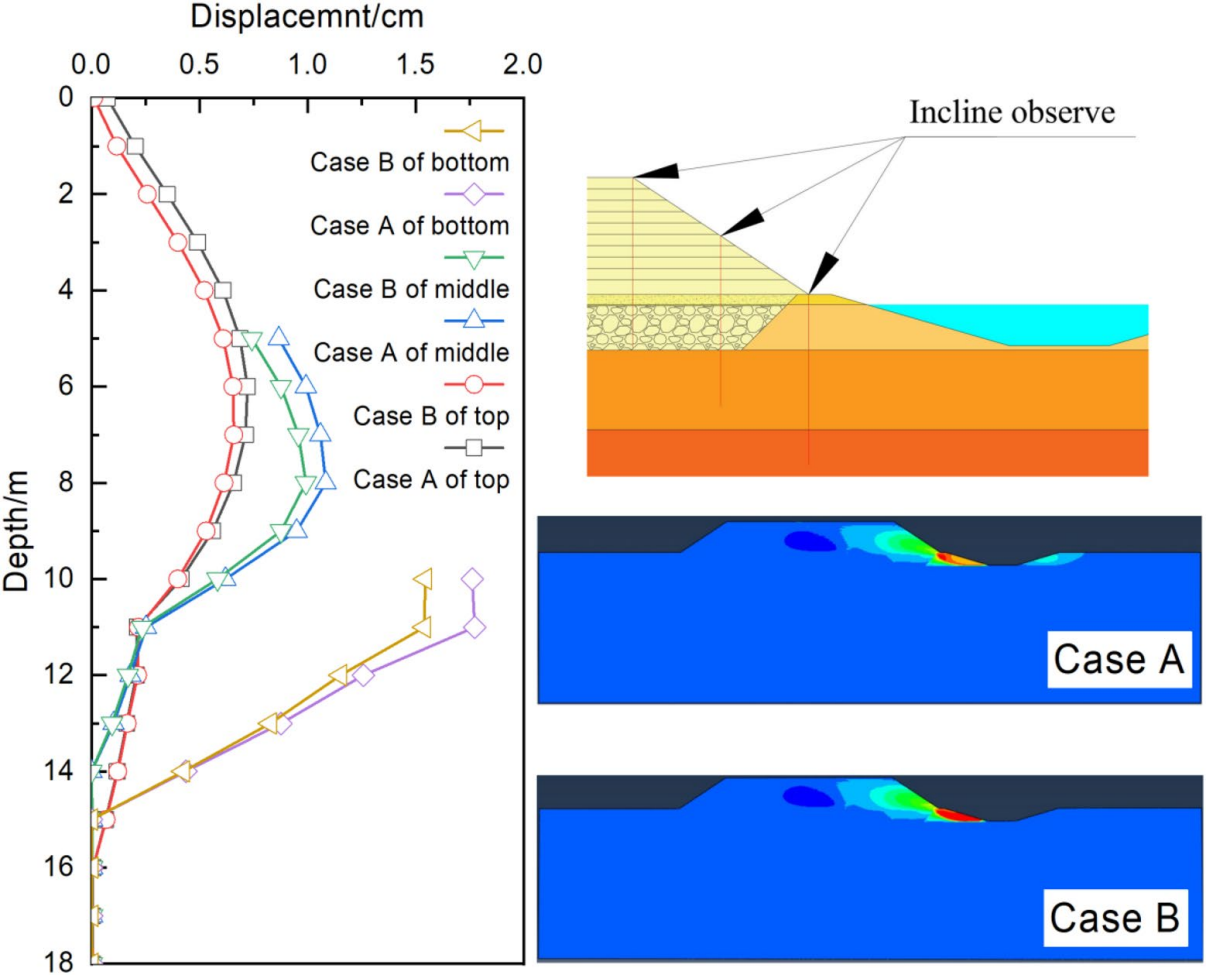
Comparison of the final slope displacements.

The displacement and cloud chart of the embankment slope are displayed in Fig. 7. It is clear that the embankment settlement had the most significant influence on the displacement at the slope top and the influence range reached almost 15 m. The embankment settlement also had a great impact on the displacement at the middle of the slope (depth = 9 m), whereas it had little influence on the displacement at the slope bottom (depth = 5 m). The influence depth of the new widening project was about 5 m because the height of the embankment filling was 10.3 m.

In Case A, the maximum displacement of the slope bottom was 1.76 cm, the minimum displacement at the slope top was 0.72 cm, and the displacement at the middle of the slope was 1.08 cm. The displacement at the slope bottom was about 2.5 times higher than that at the slope top. Due to the unconstrained embankment of the top and bottom of the slope, the soil slid outward in the process of self-weight consolidation. In addition, the soil self-weight at the slope bottom was much higher than that at the slope top, and the load horizontally deformed the foundation. Therefore, the displacement at the slope bottom was much higher than that at the slope top.

In Case B, the maximum displacements at the top, middle, and bottom of the slope were 0.66 cm, 0.99 cm, and 1.54 cm, respectively, which were 1.09, 1.09, and 1.14 times lower than those in Case A, respectively. Water pumping from the pond reduced the underground water level; thus, the soil got consolidated in the vertical and horizontal directions and led to embankment settlement and slope displacements. According to the numerical analysis results, the no-pumping condition was adopted during the construction of the widening section.

## Monitoring and evaluation

### Monitoring method and layout

**Figure 8:**
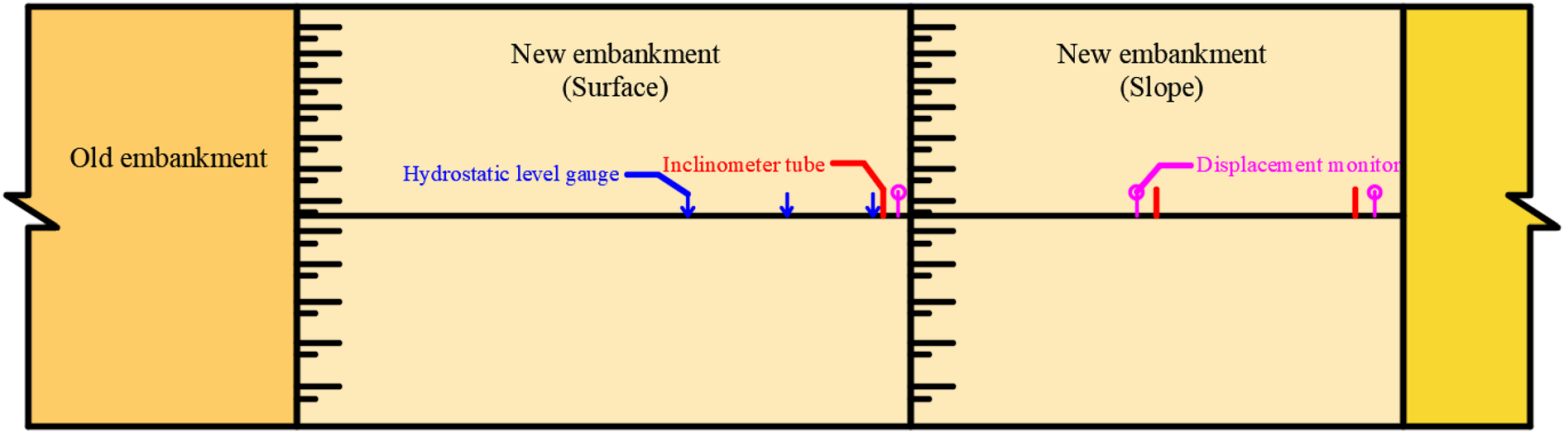
Schematic replacement of the new embankment monitoring instrument arrangement.

**Figure 9:**
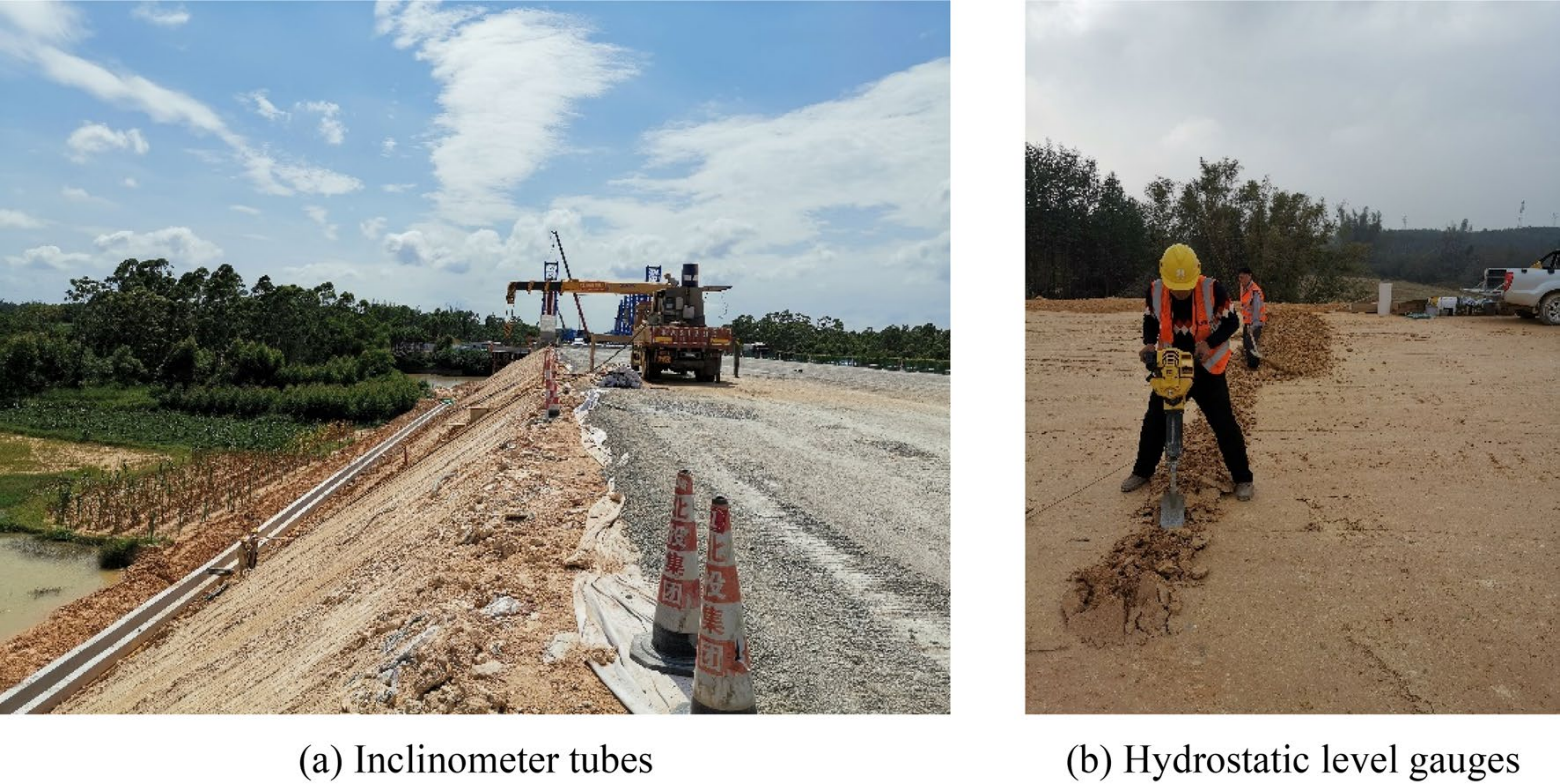
On-site monitoring instrument installation.

Settlement monitoring was carried out on the embankment surface, whereas inclination monitoring was executed on the embankment slope. Hydrostatic level gauges and inclinometer tubes were used in settlement monitoring and inclination monitoring, respectively. The hydrostatic level gauges were placed along the central line of the new embankment and the road border at a depth of about 1 m (10 dm). Three inclinometer tubes were placed at the top, middle, and bottom of the embankment slope at the depths of 14.5 m, 10 m, and 9.5 m, respectively. The monitoring equipment layout and installation processes are exhibited in Figs.8 and 9, respectively.

### Weak foundation treatment

Original embankments can be reinforced by grouting. The settlement deformation of new roads should be strictly controlled, especially in soft foundations and at junction sections of filling and excavation. The studied section has passed through the farmland and low-lying areas of the river network, and most of these areas are soft soil embankments; thus, the stability of the embankment is seriously harmed. Due to poor drainage conditions, the embankment can easily slide during highway construction.

According to the different characteristics of each section of weak foundations, the following measures are recommended: (a) The replacement method should be adopted for shallow soft soil embankments and (b) composite foundations should be used for deep soft soil embankments. When the replacement depth exceeds one meter, temporary protective measures should be taken to ensure the stability of old road embankments. Considering the economy and effectiveness of the expressway widening project, the weak foundation treatment method was used as the replacement method.

**Figure 10:**
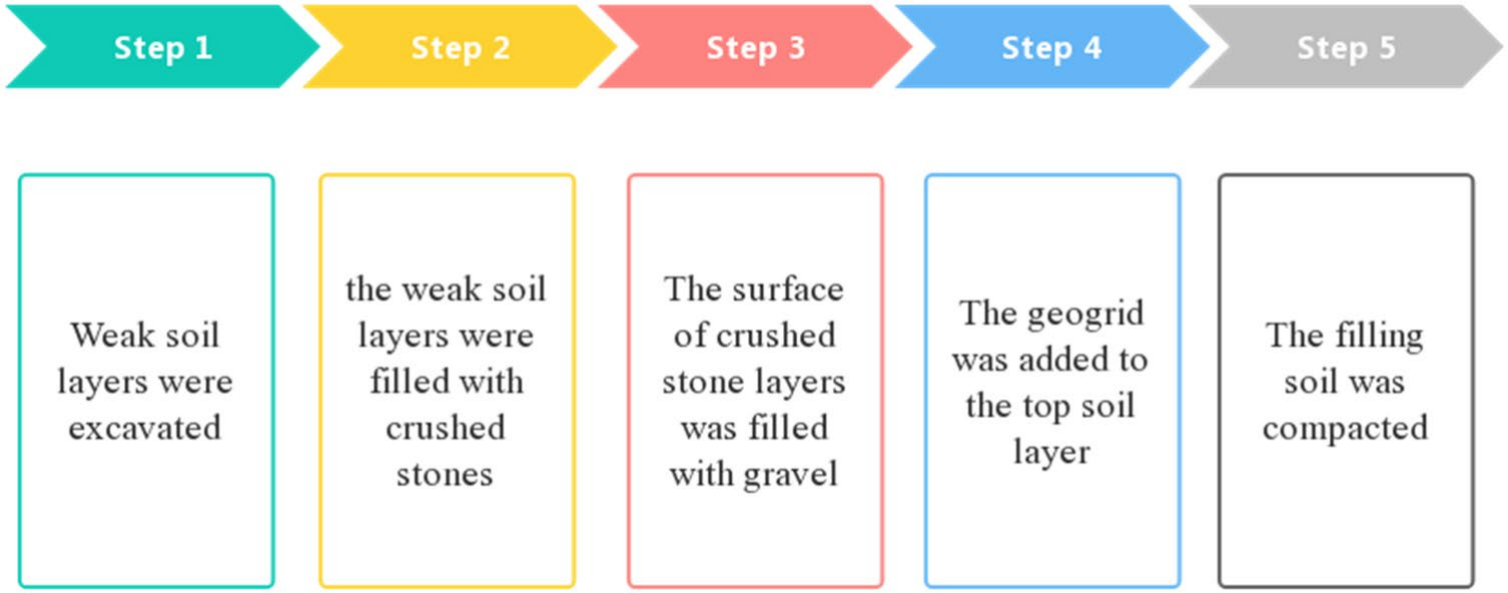
Steps of the replacement process.

**Figure 11:**
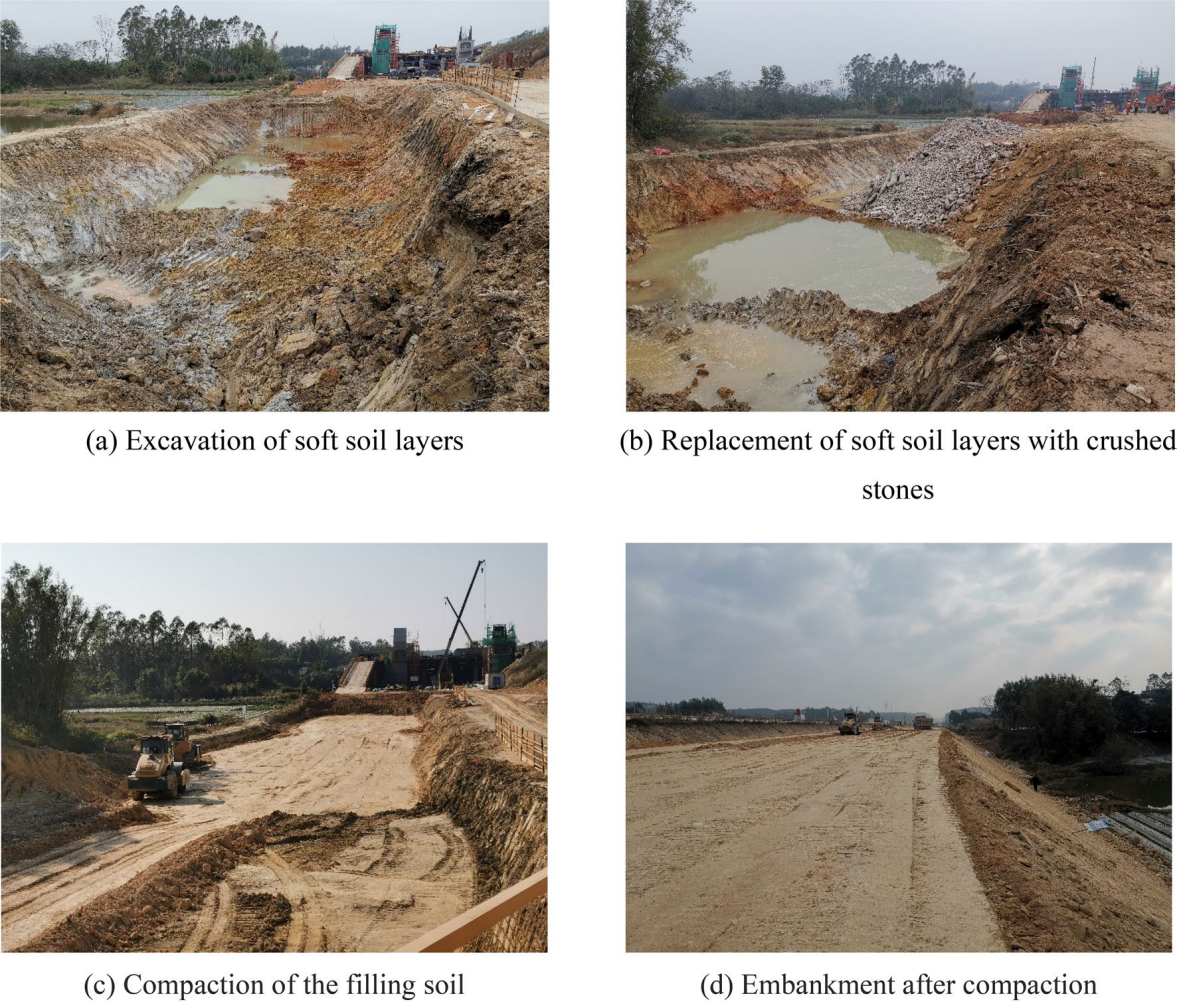
Weak soil replacement process.

Crushed stones (compressive strength = > 5 MPa) and gravel (soil content = < 3% and permeability coefficient = 0.06–0.006 cm/s) were used as the replaced material and the cushion material respectively. The height of the geogrid was > 6 m, and its parameters (Fig. 1) were tested in the lab according to the GB/T 17689–2008 standard^44^. Five steps were carried out in the replacement method (Fig. 10): a. Weak soil layers were excavated; b. The weak soil layers were filled with crushed stones. c. The surface of crushed stone layers was filled with gravel. d. The geogrid was added to the top soil layer. e. The filling soil was compacted. The weak soil replacement process is displayed in Fig. 11.

### Deformation analysis of the embankment

#### Settlement analysis

**Figure 12:**
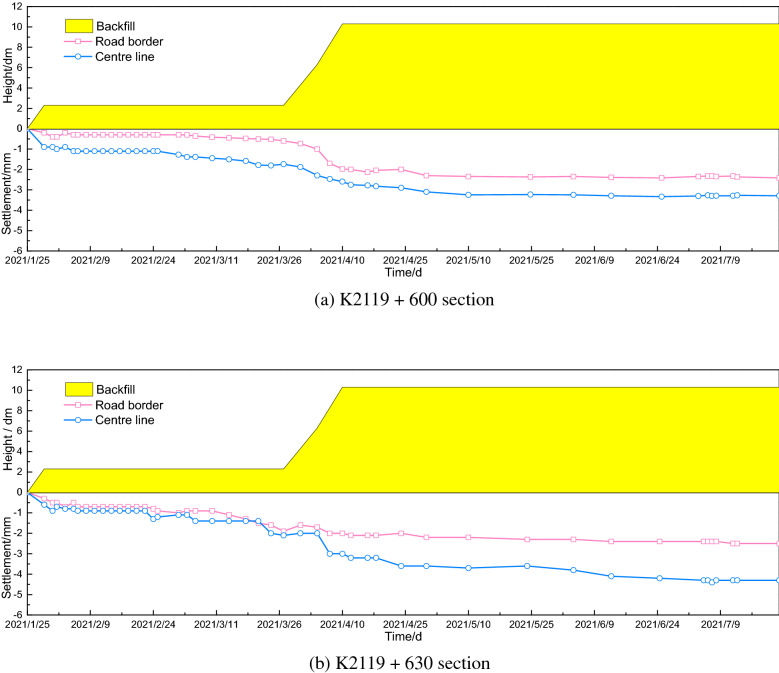
Settlement of the embankment.

The road borders and central line settlements of the K2119 + 600 and K2119 + 630 sections are presented in Fig. 12. The settlement increased with the height of the newly filled embankment. In the K2119 + 600 section, the final settlements were 4.3 mm and 2.5 mm in the central line and on the road border, respectively, and the corresponding numerical analysis results were 4.6 mm and 4.5 mm, respectively. In the K2119 + 630 section, the final settlements were 3.3 mm and 2.4 mm in the central line and on the road border, respectively; thus, the settlement difference between the central line and the road border was 0.9 mm. Therefore, it is evident that the central line had a larger settlement than the road border, implying that cracks would occur in the central line of the new embankment because the weak foundation area could increase the uneven settlement.

#### Displacement of the embankment slope

**Figure 13:**
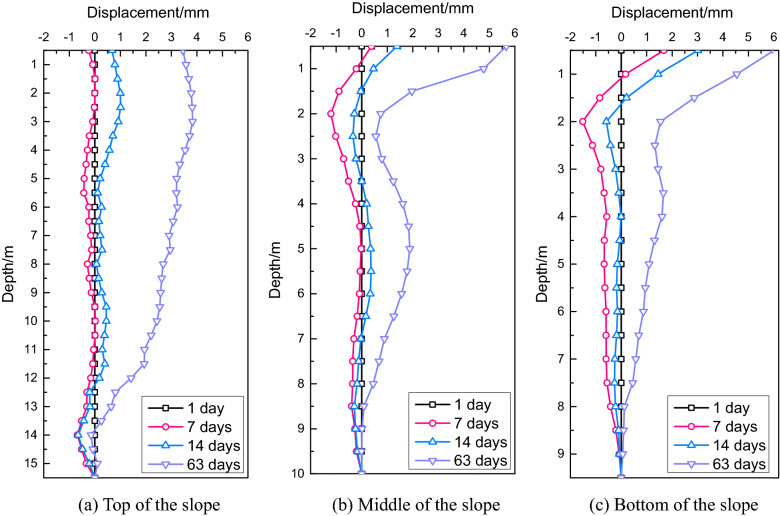
Slope displacements in the K2119 + 600 section.

**Figure 14:**
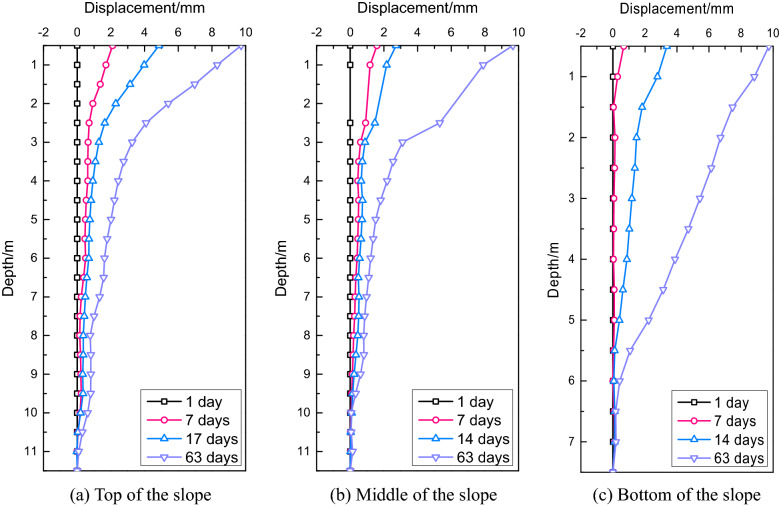
Slope displacements in the K2119 + 630 section.

The displacements of the embankment slope in the K2119 + 600 and K2119 + 630 sections are presented in Figs.13 and 14, respectively. In the K2119 + 600 section, the maximum displacements at the top, middle, and bottom of the slope were 3.72 mm, 5.67 mm, and 6.01 mm, respectively, and in the K2119 + 630 section, the corresponding values were 9.71 mm, 9.66 mm, and 9.73 mm, respectively. The settlement differences between the test and numerical values were 2.88 mm at the top, 4.23 mm at the middle, and 9.39 mm at the bottom of the slope.

Generally, embankment slopes are not confined by surrounding soil layers; thus, the displacement at the slope top is smaller than that at the slope bottom. In this project, the pond slope experienced significant deformation (Fig. 7); thus, the slope soil could not provide sufficient passive soil pressure. In addition, the water in the pond also softened the slope soil. Therefore, the displacement at the slope bottom in the new embankment was larger than that at the slope top.

### Evaluation

The fundamental problem of this widening project was the controlling of the settlement and displacement of the new and old embankments. The old embankment has been consolidated for an extended period, and the settlement is much larger than the new embankment. The new embankment is filled over a soft soil foundation. Moreover, the weak foundation is located near a pond; thus, water continually seeps into the soft soil. If the weak foundation is not reinforced, the uneven settlement may cause cracks in the new embankment. According to the drill hole test results, the old embankment has a small settlement, whereas the weak foundation has a large settlement, which can cause severe accidents in this area. Considering the soft soil volume, the replacement method has been recommended for the foundation treatment in this section. The replacement method is an effective method for shallow and weak foundations. Bi^45^ compared the costs of weak foundation treatments by the deep mixing method and the replacement method and noticed that the cost of the replacement method was about 2/3 less than that of the deep mixing method for a treatment area of about 20,000 m^2^ with depths of 1.8–3.3 m.

The water in the pond had a significant influence on the deformation of the embankment. Water pumping caused the settlement and displacement of the embankment. According to the simulation results, the pumped water increased the settlement by more than five times increase and also increased the displacement by nearly 1.1 times. In addition, as water pumping reduced the underground water level of the foundation, the floating weight increased the load on the soil; thus, the settlement increased.

**Figure 15:**
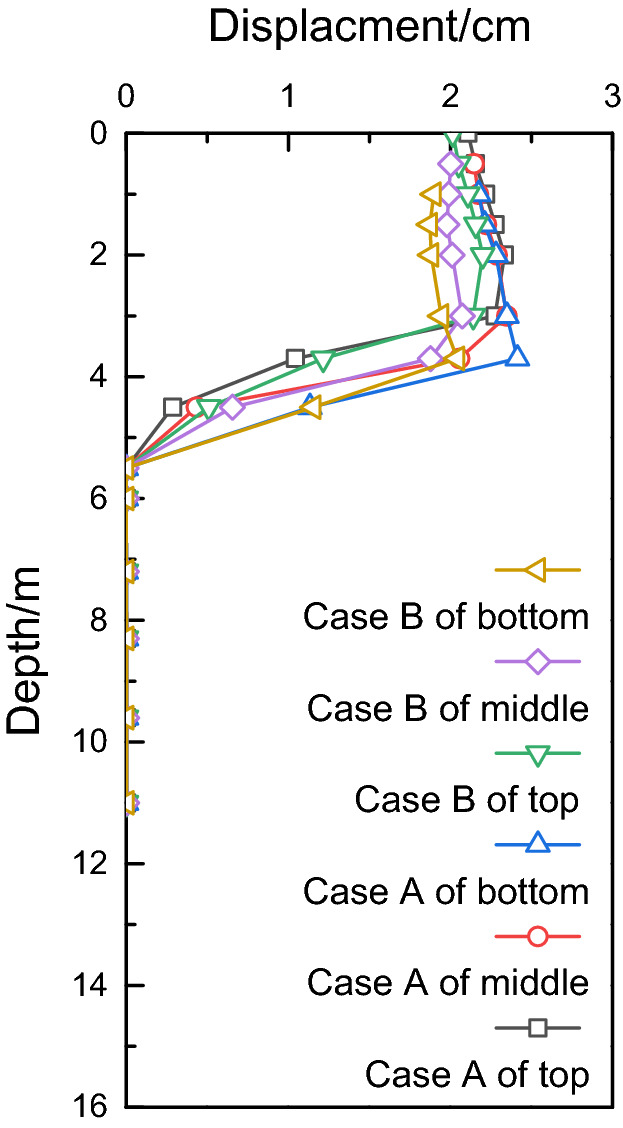
Displacements of the pond slope.

**Figure 16:**
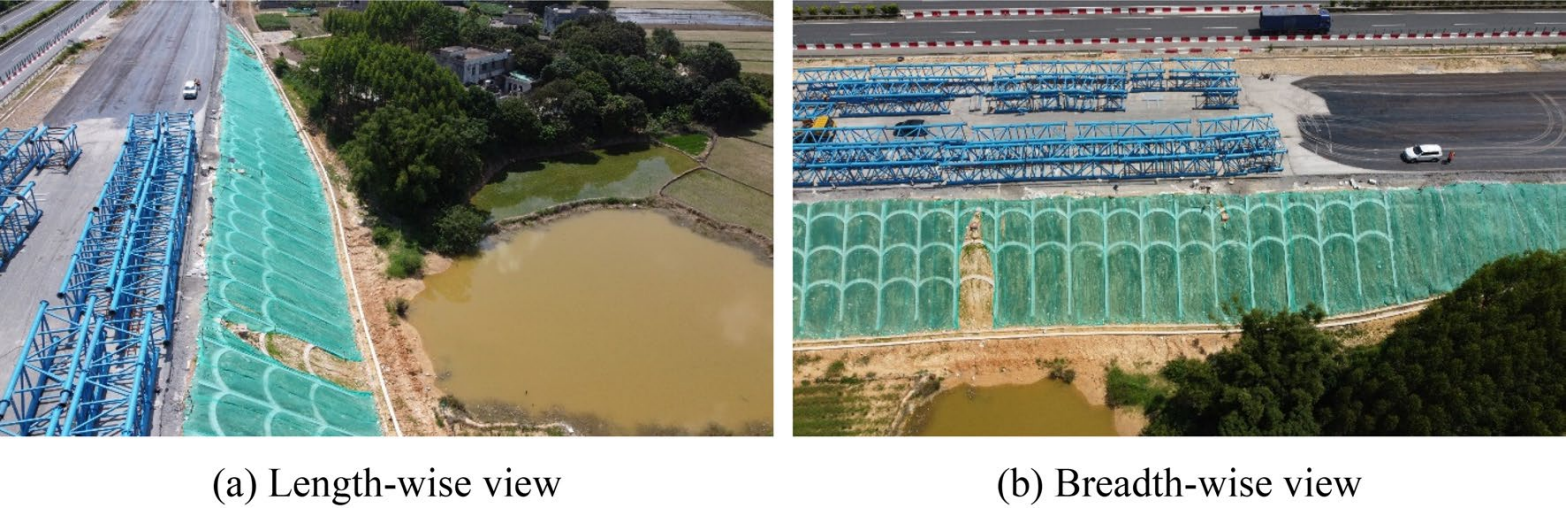
Bird’s eye views of the finished project.

Water pumping also affected the stability of the pond slope (Fig. 15). Both the left and right sides of the pond slope deformed gradually, leading to the settlement of the embankment. After the water was pumped, the new embankment had two steps; however, as the lower step (pond slope) was not treated, it more easily slid to bear the weight of the upper step. The finished widening project is presented in Fig. 16. The new embankment became stable after three months of its construction, and it still has no cracks and a prominent settlement.

## Conclusions

In order to explore the influences of underground water and weak foundation on an expressway widening project near a pond in the Qinbei section, two cases of numerical modeling under pumping and no-pumping conditions were executed. The main findings of this work are presented below.

1. The uneven settlement caused net cracks, longitude cracks, and heavy settlement in the old embankment. The drainage and slope of the foundation got affected and reduced the stability of the embankment.
2. Considering the economy and effectiveness of the project, the replacement method was recommended for the weak foundation reinforcement. The numerical analysis and on-site motoring results revealed that the slight settlement of the embankment after the replacement method had a positive effect on the soft soil foundation.
3. Water pumping significantly affected the settlement of the embankment. Heavy settlement and displacement occurred in the new embankment because water pumping caused seepage in the foundation and increased the effective stress of the soil, making the soil more consolidated under the load.
4. The pond slope should be reinforced before the construction of the embankment. The displacement of the slope top was smaller than that of the slope bottom because the pond slope was not confined by the surrounding soil; thus, the slope soil could not provide sufficient passive soil pressure and easily slid.

## Data Availability

The data used and/or analyzed in the current study are available from the corresponding author upon reasonable request.
